# Long-term inhibition of ferritin2 synthesis in trophocytes and oenocytes by ferritin2 double-stranded RNA ingestion to investigate the mechanisms of magnetoreception in honey bees (*Apis mellifera*)

**DOI:** 10.1371/journal.pone.0256341

**Published:** 2021-08-19

**Authors:** Chin-Yuan Hsu, Yu-Ting Weng

**Affiliations:** 1 Department of Biomedical Sciences, College of Medicine, Chang Gung University, Tao-Yuan, Taiwan; 2 Graduate Institute of Biomedical Sciences, College of Medicine, Chang Gung University, Tao-Yuan, Taiwan; 3 Institute of Stem Cell and Translational Cancer Research, Lin-Kou Medical Center, Chang Gung Memorial Hospital, Linkou, Taiwan; King Khalid University, SAUDI ARABIA

## Abstract

Behavioral studies indicate that honey bees (*Apis mellifera*) have a capacity for magnetoreception and superparamagnetic magnetite is suggested to be a magnetoreceptor. The long-term inhibition of magnetite formation can be employed to explore the bee’s magnetoreception. A recent study shows that magnetite formation, *ferritin2* messenger RNA (mRNA) expression, and the protein synthesis of *ferritin2* in trophocytes and oenocytes were all inhibited by a single injection of *ferritin2* double-stranded RNA (dsRNA) into the hemolymph of honey bees but how to maintain this knockdown of *ferritin2* for the long-term is unknown. In this study, we injected *ferritin2* dsRNA into the hemolymph of worker bees three times every six days to maintain long-term inhibition; however, multi-microinjections accelerated the death of the bees. To overcome this problem, we further reared newly emerged worker bees daily with *ferritin2* dsRNA throughout their lives, demonstrating no impact on their lifespans. Follow-up assays showed that the mRNA expression and protein synthesis of *ferritin2* were persistently inhibited. These findings verified that daily *ferritin2* dsRNA ingestion not only displays the long-term inhibition of mRNA expression and protein synthesis of *ferritin2*, but also did not damage the bees. This method of long-term inhibition can be used in behavioral studies of magnetoreception in honey bees.

## Introduction

Magnetoreception is a sense that allows animals to create magnetic maps for navigation and positioning using the Earth’s magnetic field. Honey bees (*A*. *mellifera*) have the capacity of magnetoreception based on behavioral evidence. Bees’ comb construction and homing behaviors are affected by the addition of a magnetic field [1–3]. Bees can detect small static intensity fluctuations at a level of 26 nT (nanotesla) against the earth-strength magnetic field [4, 5]. 26 nT is the intensity of the magnetic field at a current of 2 x 10^−5^ ampere in a laboratory training apparatus [4]. At this low current, the trained bees can still meet the behavioral response criteria [4]. Bees can detect localized anomalies in a magnetic field [6, 7]. Bees can detect magnetic stimuli, and the signal is sent by the ventral nerve cord [8].

The discovery of superparamagnetic magnetite in the iron granules (IGs) of iron deposition vesicles (IDVs) of trophocytes supports the behavioral evidence of magnetoreception in honey bees [9, 10]. A magnetic field causes the conformation changes of IGs resulting in the fluctuation of cytoskeletons on IDVs, which are used to establish a magnetic map during orientation flights [10]. Trophocytes are located in the fat bodies of the abdomen of honey bees [11] and the magnetic sensing signal is transferred through the ventral nerve cord [8]. IGs, therefore, are proposed to be the magnetoreceptor in honey bees [10].

IGs are formed from the aggregation of 7.5-nm diameter iron particles in the center of IDVs of trophocytes [11]. An actin-myosin-ferritin transporter system including actin, myosin, ferritin, and ATP synthase in IDVs participates in the formation of IGs [12]. Ferritin is a hollow globular protein containing heavy chains and light chains. Heavy chains called ferritin1 are important for Fe^+2^ oxidation and have a relationship with the transportation of 7.5-nm diameter iron particles [13, 14]. Light chains called ferritin2 assist in core formation and participate in the formation of 7.5-nm diameter iron particles [13, 14].

RNA interference (RNAi)-mediated gene knockdown has been used to knock down *vitellogenin*, *octopamine receptor*, *DNA methyl-transferase*, *insulin receptor substrate*, *tyramine receptor 1*, *naked cuticle*, and *transferrin* by double-stranded RNA (dsRNA) injection or ingestion in adult honey bees [15–22]. Recently, we have successfully knocked down *ferritin2* and *ferritin1* by the one injection of dsRNA into the hemolymph of honey bees [14]. The mRNA expression and protein synthesis of *ferritin2* and the formation of magnetite were inhibited [14]. The one injection of *ferritin2* dsRNA shows an inhibitory effect, but its inhibitory effect did not last long enough for behavioral studies.

Finding a procedure for *ferritin2* RNAi that not only can have a long-term knockdown effect throughout bee’s lives but also does not damage the bees is important for behavioral studies to explore the magnetoreception of honey bees.

## Materials and methods

### Ethics statement

The experimental honey bees (*A*. *mellifera*) containing pupae from different colonies were purchased from a single commercial breeder (Hsinchu, Taiwan) and were kept in the Department of Biomedical Sciences, Chang Gung University, Taiwan. Although honey bees are neither an endangered nor protected species, we comply with the regulations of the laboratory animal care and use committee of Chang Gung University.

### The preparation of dsRNA toward *ferritin2* and *green fluorescent protein (GFP)*

The primers were designed according to the nucleotide sequences available in GenBank: *ferritin2* (Fer2LCH) (XM_624073.4): forward 5’-ATTTTTGGCAACTGCCTCTG-3’, reverse 5’-ATTCTCGAACACGGTCTGCT-3’; *GFP*: forward 5’-GAGATACCCAGATCAT-3’, reverse 5’-GATGATATTCACCACTT-3’. Primers were fused with T7 promoter sequence (5’- TAATACGACTCACTATAGGGCGA-3’). Total RNA was isolated from the trophocytes and oenocytes of three worker bees at 3 days after adult emergence using TRIzol (15596018; Invitrogen, Carlsbad, CA, USA) following the manufacturer’s instructions. The double-stranded DNA (dsDNA) was synthesized by using Superscript III First-Strand Synthesis System for reverse transcriptase-polymerase chain reaction (RT-PCR) (18080–051; Invitrogen, Carlsbad, CA, USA). Briefly, the synthesis of dsDNA had two steps: one was to synthesize the first-strand complementary DNA (cDNA) by reverse transcriptase, and the other was to synthesize dsDNA from the cDNA by PCR. We used 1 μg of total RNA and followed the manufacturer’s instructions to synthesize cDNA (18080–051; Invitrogen). For synthesizing dsDNA, the 50 μl PCR mixture contained the following: 2 μl cDNA, 1.5 μl forward primer, 1.5 μl reverse primer, 25 μl PCR master mix, and 20 μl H_2_O. The PCR program was 95°C for 3 min, followed by 36 cycles of 95°C for 30 s, 53°C for 35 s, and 72°C for 45 s, and then 72°C for 10 min in a TProfessional Thermocycler (070–851; Biometra, Goettingen, Germany). The dsDNA was purified by QIA Quick Gel Extraction Kit (28704, Qiagen, Valencia, CA, USA). The 2 μl of dsDNA, 600 ng/μl, was transformed into *E*. *coli* by using Topo TA Cloning Kit for sequencing (450030, Invitrogen, Carlsbad, CA, USA). The plasmid was isolated with QIAprep Spin Miniprep Kit (27104, Qiagen, Valencia, CA, USA). The dsDNA was amplified by PCR using the T7 primers. The 50 μl PCR mixture contained the following: 1 μl plasmid DNA, 1.5 μl forward primer, 1.5 μl reverse primer, 25 μl PCR master mix, and 21 μl H_2_O. The PCR program was 95°C for 3 min, followed by 36 cycles of 95°C for 30 s, 55°C for 35 s, 72°C for 45 s, and then 72°C for 10 min in a TProfessional Thermocycler (070–851; Biometra). After PCR amplification, gel electrophoresis via 1.0% agarose gels was performed to verify the expected target. The PCR product was purified by QIA Quick Gel Extraction Kit (28704, Qiagen) for dsRNA synthesis. The dsRNA was synthesized from PCR product and purified by using AmpliScribe^TM^ T7-Flash^TM^ Transcription Kit (ASF3257, Epicentre Biotechnologies, Madison, WI, USA) following the manufacturer’s instructions. The 20 μl reactive mixture contained the following: 6.8 μl the PCR product, linearized template DNA, 2 μl AmpliScribe^TM^ T7-Flash 10X reaction buffer, 1.8 μl ATP, 1.8 μl CTP, 1.8 μl GTP, 1.8 μl UTP, 2 μl DTT, and 2 μl AmpliScribe^TM^ T7-Flash enzyme solution. Gel electrophoresis via 1.0% agarose gels was performed to verify the expected target. The dsRNA was diluted with nuclease-free water to a final concentration of 5 μg/μl [14, 21–23].

### The multi-microinjections of *ferritin2* and DEPC water

The brood combs of honey bees (*A*. *mellifera*) containing pupae from the source colony were purchased from a single commercial breeder (Hsinchu, Taiwan) and transferred to an incubator (34°C, 75% relative humidity) [24]. Seventy newly emerged worker bees were collected in a cage (15x10x12 cm) and put into a 34°C thermostat (NK system, Nippon, Japan). Worker bees were fed honey and fresh pollen grains mixed with honey (3:1) every day [24]. For microinjection, worker bees were immobilized on a disc of bee wax with two crossed metal needles at room temperature (25 ± 1°C). The bees were injected with 1 μl nuclease-free water (diethylpyrocarbonate (DEPC)-treated water) (DEPC water group) or 1 μl *ferritin2* dsRNA solution (5 μg/μl) (Fer2 RNAi group) with a microinjector (FemtoJet, Eppendorf, Hamburg, Germany). Worker bees without microinjection were the control group. Microinjection was performed on the dorsum of the abdomen between the 1st and 2nd abdominal segment with glass needles. Individuals showing hemolymph leakage after microinjection were discarded. Successfully injected bees were housed in a cage (15x10x12 cm) for 1 h before moving into an incubator set to 34°C (NK system, Nippon, Japan) [14]. Microinjection was carried out three times every 6 days and the number of survival bees was calculated at 1, 7, 13, and 19 days. This experiment was replicated three times and two hundred ten worker bees in total were used in each group.

### The feeding of *ferritin2* dsRNA, *GFP* dsRNA, or DEPC water and survivorship

The collection of worker bees was mentioned above. Fifty newly emerged worker bees were collected in a cage and put into a 34°C thermostat. Each worker bee of the control group, the DEPC group, the GFP RNAi group, and the Fer2 RNAi group was fed 30μl honey with 1.5μl ddH_2_O and commercial fresh pollen grains mixed with honey, 30μl honey with 1.5μl DEPC-treated water and fresh pollen grains mixed with honey, 30μl honey with 1.5μl of 5 μg/μl *GFP* dsRNA and fresh pollen grains mixed with honey, and 30μl honey with 1.5μl of 5 μg/μl *ferritin2* dsRNA and fresh pollen grains mixed with honey every day, respectively. Survivorship of worker bees was recorded every day. The survivorship, mean lifespan, and maximum lifespan were analyzed by SPSS software (version 10, SPSS, Chicago, IL, USA) [24]. This experiment was replicated four times and two hundred worker bees in total were used in each group.

### Quantitative real-time polymerase chain reaction (qPCR) analyses

Trophocytes and oenocytes were isolated from two worker bees reared with ddH_2_O (control group), with *GFP* dsRNA (GFP RNAi group), with *ferritin2* dsRNA (Fer2 RNAi group) at 3, 7, 11, 15, and 20 days after adult emergence. Worker bees of each group were dissected with scissors and their abdominal trophocytes and oenocytes were detached from the cuticle using a knife in honey bee saline (156.4 mM NaCl, 2.7 mM KCl, 1.8 mM CaCl_2_, 22.2 mM glucose, pH 7.3) and collected by centrifugation [10]. Total RNA was extracted from these cells using Trizol^®^ Reagent (15596018; Invitrogen, CA, USA). RNA concentration and quality were determined using a Synergy^TM^ HT multi-mode microplate reader (7091000; BioTek). The complementary DNA (cDNA) synthesis was performed using an iScript™ cDNA Synthesis Kit (170–8891; Bio-Rad Laboratories, CA, USA). Amplification was performed in a TProfessional Thermocycler (070–851; Biometra). Each reaction contained 1 μg of total RNA in a 20 μl reaction volume. The qPCR was performed using a CFX connect RT-PCR detection system (Bio-Rad Laboratories, CA, USA) and each reaction contained 0.5 μl of 10 μM of each primer, 12.5 μl of SYBR Green (170–8882; Bio-Rad Laboratories), 1 μl of diluted cDNA, and 10.5 μl of ddH_2_O in a final volume of 25 μl [14]. Primer sequences were noted above. The *β-actin* gene was used as a reference gene [25]. The primers were designed according to the nucleotide sequences available in GenBank: *β-actin* (AB023025): forward 5’-ATGCCAACACTGTCCTTTCTGG-3’, reverse 5’-GACCCACCAATCCATACGGA-3’. The PCR program was 95°C for 3 min, followed by 39 cycles of denaturation at 95°C for 10 s and annealing at 60°C for 30 s. All samples were run in quadruplicate. The relative expression levels of genes were calculated using the 2^−ΔΔCt^ method [26]. Ten replicates were performed, and twenty worker bees in total were used in each group.

### Western blotting

Trophocytes and oenocytes were isolated from two worker bees reared with ddH_2_O (control group), with *GFP* dsRNA (GFP RNAi group), with *ferritin2* dsRNA (Fer2 RNAi group) at 20 days after adult emergence, homogenized in 100 μl of radioimmunoprecipitation (RIPA) lysis buffer (50 mM Tris-HCl, pH 7.4, 150 mM NaCl, 5% deoxycholic acid, 0.10% NP-40, 5mM ethylenediaminetetraacetic acid (EDTA), and 0.1% sodium dodecyl sulfate (SDS)) containing protease inhibitors (11697498001; Roche Applied Science, Indianapolis, IN, USA), and centrifuged at 5,000 *g* for 10 min at 4°C. The protein concentration of the resulting supernatant was determined using a protein assay reagent (500–0006; Bio-Rad Laboratories, Hercules, CA, USA). Proteins (30 μg) from the supernatant were resolved by SDS-polyacrylamide gel electrophoresis (SDS/PAGE) on 10–15% polyacrylamide gels and transferred to polyvinylidene fluoride (PVDF) membranes. After blocking for 1h at 25°C, membranes were first incubated with primary antibodies against ferritin2 (1:1,000; produced in-house) [12] or tubulin (ab6046, 1:10,000; Abcam, Cambridge, MA, USA) and then probed with the appropriate horseradish peroxidase-conjugated secondary antibody (1:10,000). Anti-ferritin2 antibodies were produced in rabbits using peptides corresponding to the COOH-terminal region of honey bee ferritin2 (amino acids 154–172; KIHEKANKKQDSAIAHYME) [12]. Immunoreactive proteins were detected using a chemiluminescence method (PerkinElmer, Covina, CA, USA) and analyzed using Image J software (NIH, Bethesda, MA, USA). The protein expression levels were normalized to tubulin [24]. Ten replicates were performed, and twenty worker bees in total were used in each group.

### Statistical analysis

SPSS software was used for statistical analyses [24]. Differences in the mean values among the three treatment groups were determined by one-way ANOVA and by Tukey’s HSD for pairwise comparisons. A *p*-value of less than 0.05 was considered statistically significant. Survivorship was calculated using the log-rank (Mantel-Cox) method. A *p*-value of less than 0.005 was considered statistically significant.

## Results

### The multi-microinjections of dsRNA damages bees

**Fig 1:**
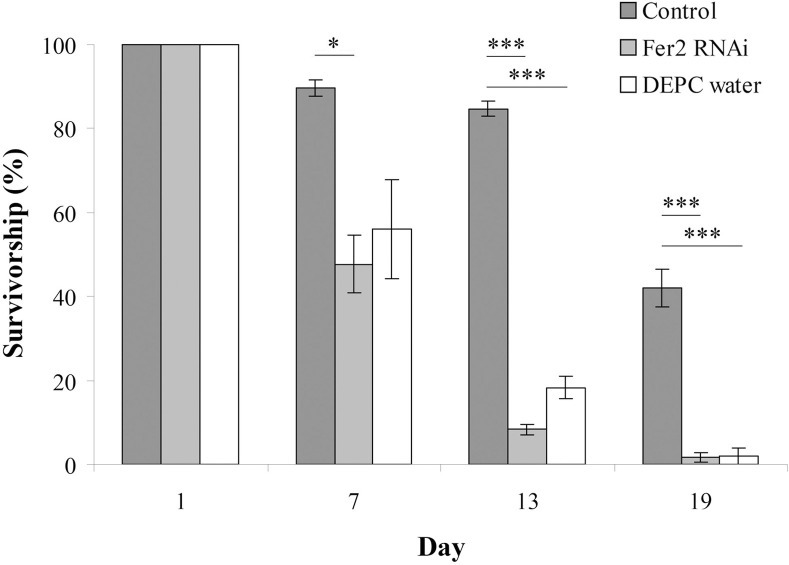
The survivorship of worker bees injected with *ferritin2* dsRNA or DEPC water at 1, 7, 13, and 19 days after adult emergence. Control, no injection. Fer2 RNAi, *ferritin2* dsRNA injection. DEPC water, DEPC water injection. The results were expressed as percentages and presented as the means at 1 day and as the means ± standard error of the means (SEMs) at 7, 13, and 19 days (*n* = 210). Asterisks indicate statistical significance (*P < 0.05; ***P< 0.001; one-way ANOVA).

To keep a continuous *ferritin2* RNAi knockdown effect for exploring the mechanisms of magnetoreception, we injected *ferritin2* dsRNA into the hemolymph of worker bees three times every six days. The experiments showed that the number of surviving bees decreased at 7, 13, and 19 days when they were injected with *ferritin2* dsRNA or DEPC water compared to the non-injected control (*n* = 210, *P* < 0.05; Fig 1), indicating that multi-microinjections damaged the bees.

### The survivorship of bees is not shortened by the *ferritin2* dsRNA ingestion

**Fig 2:**
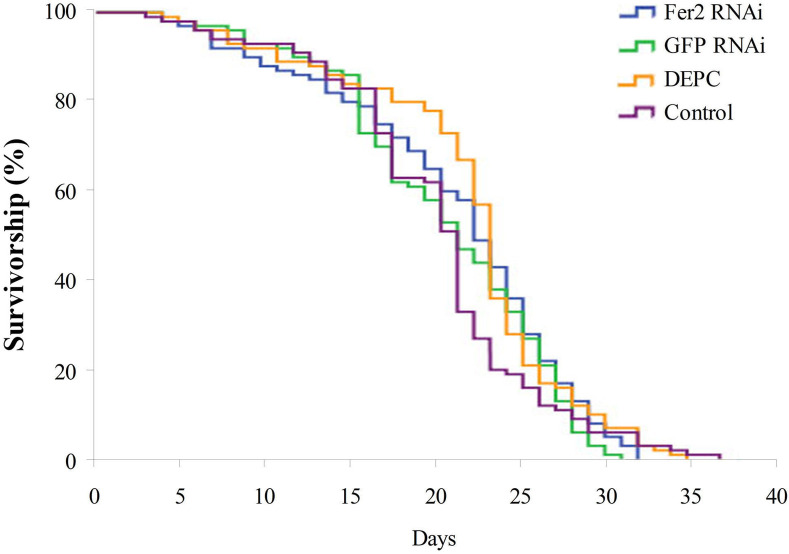
The survivorship of worker bees in the *ferritin2* RNAi, the *GFP* RNAi, the DEPC water, and the control. Fer2 RNAi, feeding with *ferritin2* dsRNA. GFP RNAi, feeding with *GFP* dsRNA. DEPC, feeding with DEPC water. Control, no additional feeding (*n* = 200; *P* = 0.149). *P*-value was calculated using the log-rank (Mantel-Cox) method.

To find a way to continuously induce the *ferritin2* RNAi knockdown effect without damaging the bees, we reared newly emerged worker bees with *ferritin2* dsRNA throughout their lives. The results revealed that the lifespan of worker bees were not shortened after feeding them *ferritin2* dsRNA compared to *GFP* dsRNA, the DEPC water, and the non-fed control (*n* = 200, *P* > 0.05; Fig 2). The mean lifespan and the maximum lifespan of worker bees reared with *ferritin2* dsRNA were not significantly different compared to those reared with *GFP* dsRNA, the DEPC water, or the control (*n* = 200, *P* > 0.05; Table 1). These results revealed that dsRNA ingestion did not damage bees.

**Table 1:** Mean and maximum lifespan of worker bees of *ferritin2* RNAi, *GFP* RNAi, DEPC water, and control.

Treatments	Mean lifespan ± SEM (days)	Maximum lifespan ± SEM (days)	N
Ferritin2 RNAi	21.75 ± 0.74	31.50 ± 0.65	200
GFP RNAi	21.25 ± 0.66	30.50 ± 0.65	200
DEPC water	22.63 ± 0.68	33.17 ± 0.95	200
Control	20.76 ± 0.66	33.50 ± 1.43	200

Maximum lifespan is the mean lifespan of the 10% of population that had the longest lifespans. N, number of worker bees analyzed. RNAi, RNA interference; GFP, green fluorescent protein; DEPC, diethylpyrocarbonate; SEM, standard error of the means.

### The mRNA expression of ferritin2 is inhibited by the *ferritin2* dsRNA ingestion

**Fig 3:**
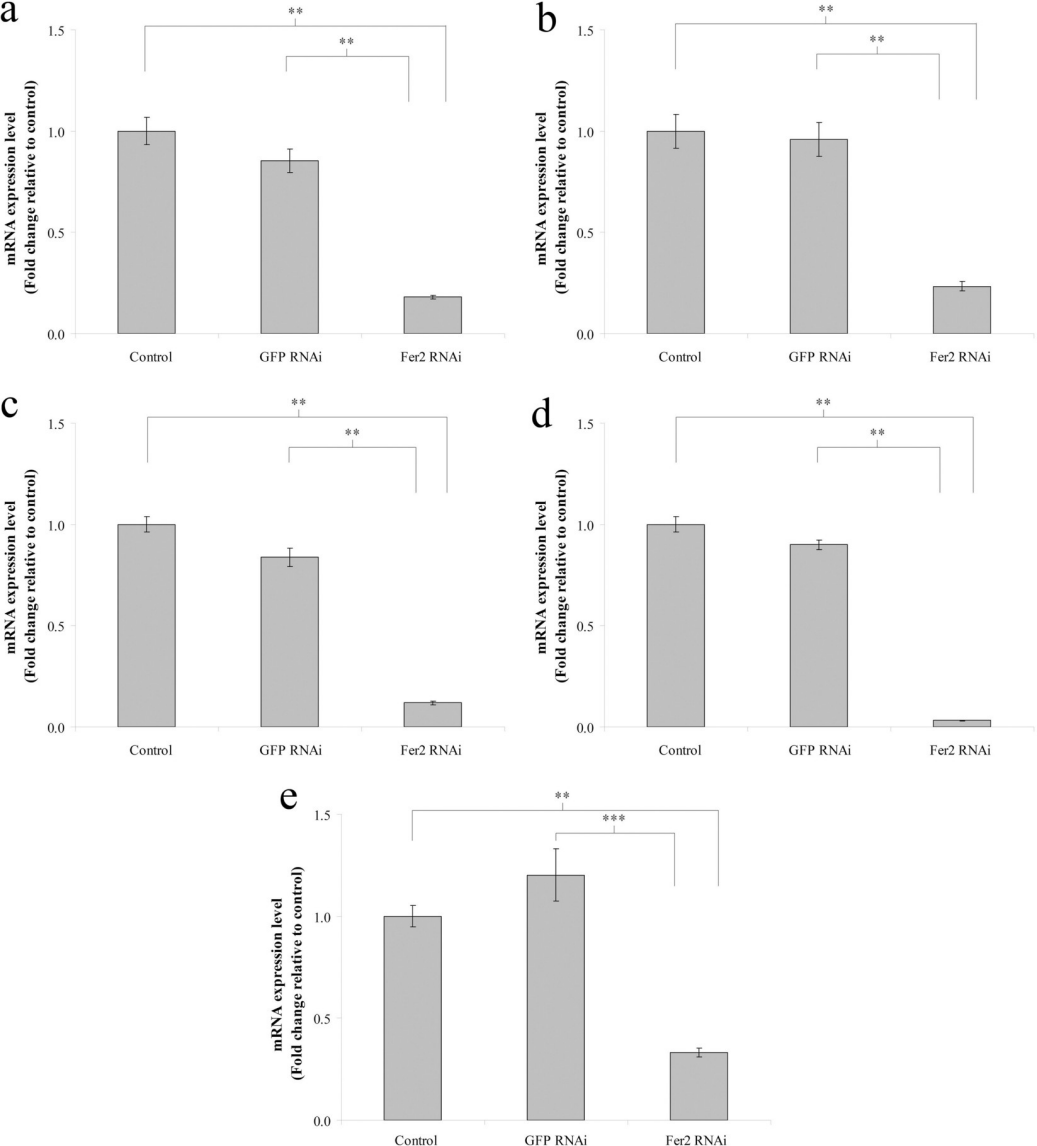
The mRNA expression of ferritin2 in trophocytes and oenocytes of worker bees at 3 (a), 7 (b), 11 (c), 15 (d), and 20 (e) days after feeding with *ferritin2* dsRNA. Fer2 RNAi, feeding with *ferritin2* dsRNA. GFP RNAi, feeding with *GFP* dsRNA. Control, feeding with water. Actin served as the loading control. The results were normalized to the control and were shown as fold changes, representing the mean ± SEMs (*n* = 10). Asterisks indicate statistical significance (**P < 0.01; ***P< 0.001; one-way ANOVA).

To evaluate the RNAi effect of *ferritin2* dsRNA ingestion, we assayed the mRNA levels of *ferritin2* in the trophocytes and oenocytes of worker bees reared with *ferritin2* or *GFP* dsRNA at 3, 7, 11, 15, and 20 days. The results showed that the mRNA levels of *ferritin2* in trophocytes and oenocytes decreased at 3, 7, 11, 15, and 20 days compared to the control or the *GFP* RNAi (*n* = 10, *P* < 0.05; Fig 3A–3E), indicating that the *ferritin2* dsRNA daily ingestion persistently suppressed *ferritin2* mRNA expression.

### The protein synthesis of ferritin2 is inhibited by the *ferritin2* dsRNA ingestion

**Fig 4:**
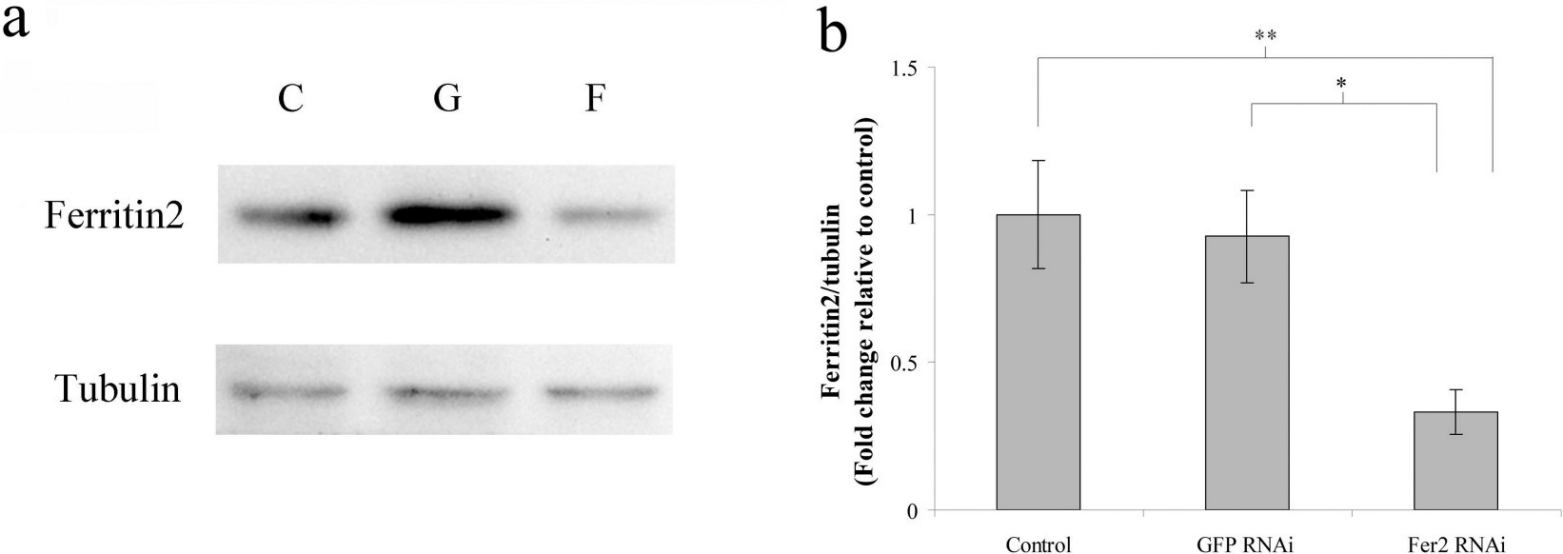
The protein levels of ferritin2 in trophocytes and oenocytes of worker bees at 20 days after feeding with *ferritin2* dsRNA. (**a**) The protein levels of ferritin2. F, feeding with *ferritin2* dsRNA. G, feeding with *GFP* dsRNA. C, feeding with water. Tubulin served as the loading control. (**b**) The results were normalized to the control and shown as fold changes, representing the mean ± SEMs (*n* = 10). Fer2 RNAi, feeding with *ferritin2* dsRNA. GFP RNAi, feeding with *GFP* dsRNA. Control, feeding with water. Asterisks indicate statistical significance (*P < 0.05; **P < 0.01; one-way ANOVA).

To verify the RNAi effect of *ferritin2* dsRNA ingestion, we assayed the protein levels of ferritin2 in the trophocytes and oenocytes of worker bees reared with *ferritin2* or *GFP* dsRNA at 20 days because worker bees reared with *ferritin2* dsRNA at 20 days were used for behavior studies. The findings revealed that the protein levels of ferritin2 in trophocytes and oenocytes declined at 20 days compared to the control and the *GFP* RNAi (Fig 4A). Statistical analyses showed that the protein levels of ferritin2 were significantly different compared to the control and the *GFP* RNAi (*n* = 10, *P* < 0.05; Fig 4B), indicating that the *ferritin2* dsRNA ingestion every day persistently suppressed the protein synthesis of ferritin2.

## Discussion

We previously injected *ferritin2* dsRNA once into the hemolymph of newly emerged worker bees and demonstrated that the mRNA expression and protein synthesis of ferritin2 and the formation of magnetite are inhibited by *ferritin2* RNAi [14]. However, maintaining a continuous *ferritin2* RNAi effect is important for further exploring the mechanism of magnetoreception. This study demonstrated that the *ferritin2* dsRNA daily ingestion persistently inhibits the mRNA expression and protein synthesis of ferritin2 without any impact on the bee’s lifespans.

### The multi-microinjections of dsRNA damages bees

Although one single injection of *ferritin2* dsRNA inhibits the mRNA expression and protein synthesis of ferritin2 and the formation of magnetite, by only keeping continuous *ferritin2* RNAi effects or long-term knockdown can this approach be used for behavioral studies to explore the mechanism of magnetoreception. For this goal, we continuously injected *ferritin2* dsRNA into the hemolymph of worker bees three times every six days; however, this type of injection damages bees leading to the decline in the survival number of bees. Similar damage is also present in the multi-microinjections of DEPC water. The most likely reason for these phenomena is that multi-microinjections result in long-term hemolymph leakage, which damages bees. This inference is supported by previous studies showing that dsRNA injection into hemolymph causes hemolymph leakage [14, 15, 27]. Therefore, long-term knockdown through multi-microinjections are not feasible for further behavioral studies.

### The lifespan of bees is not shortened by the *ferritin2* dsRNA ingestion

To overcome the damage of multi-microinjections, we fed newly emerged worker bees with *ferritin2* dsRNA throughout their lives. The lifespan of bees reared with *ferritin2* dsRNA was similar to that of the *GFP* RNAi, the DEPC water, and the control. This phenomenon reveals that the *ferritin2* dsRNA ingestion does not damage bees. This statement is consistent with previous studies indicating that the feeding of *naked cuticle*-dsRNA in *Nosema ceranae*-infected bees extends the lifespan of bees and improves the overall health of bees [21] and the feeding of deformed wing virus (DWV)-dsRNA does not affect bee’s survival [28]. The most likely reason is that dsRNA has no toxicity for honey bees [29] or that dsRNA does not completely knock down ferritin2 to affect the physiology of bees [14]. Therefore, the *ferritin2* dsRNA ingestion has a potential for utilization in behavior studies of magnetoreception of honey bees. The ingested *ferritin2* dsRNA was exported into hemolymph where *ferritin2* dsRNA was associated with proteins, forming extracellular ribonucleoprotein complexes. Once *ferritin2* dsRNA in hemolymph was taken up by trophocytes, it was cut into double-stranded small interference RNAs by Dicer in the cytoplasm to perform the gene silencing [20, 30, 31].

### The mRNA expression and protein synthesis of ferritin2 are inhibited by the *ferritin2* dsRNA ingestion

To determine the *ferritin2* RNAi effect of *ferritin2* dsRNA ingestion, the mRNA expression and protein synthesis levels of ferritin2 were assayed. The mRNA expression and protein synthesis of ferritin2 in trophocytes and oenocytes decreased after the *ferritin2* dsRNA ingestion, demonstrating that the feeding of *ferritin2* dsRNA throughout the bee’s lives inhibits the mRNA expression and protein synthesis of ferritin2. The RNAi effect of *ferritin2* ingestion is similar to the one from the injection RNAi effect that inhibits the mRNA expression and protein synthesis of ferritin2 [14]; furthermore, *ferritin2* dsRNA ingestion has a long-term knockdown effect. The RNAi effect of *ferritin2* daily ingestion corresponds with previous studies showing that the feeding of Israeli acute paralysis virus (IAPV)-dsRNA lowers IAPV level and prevents bee’s mortality [25] and the feeding of DWV-dsRNA reduces wing deformity [28].

Notwithstanding the mRNA expression and protein synthesis of ferritin2 were inhibited by *ferritin2* dsRNA ingestion, the lifespan of *ferritin2* RNAi knockdown honey bees was not shortened compared to the control bees. The most likely reason is that *ferritin2* RNAi does not completely knock down *ferritin2*, resulting in the partial synthesis of ferritin2 protein, which can perform the normal physiological function in bees. This phenomenon corresponds to a previous study indicating that vitellogenin (Vg) RNAi decreases the mRNA expression of Vg but does not shorten the lifespan of honey bees [23].

## Conclusions

We injected *ferritin2* dsRNA into the hemolymph of worker bees three times every six days to maintain long-term inhibition of *ferritin2*; however, multi-microinjections accelerated the bees’ death. The most likely reason is the leakage of hemolymph after injection. Therefore, this method is not feasible for long-term inhibition of ferritin2 synthesis. By contrast, newly emerged worker bees that are daily reared with *ferritin2* dsRNA throughout their lives do not display a shortened lifespan compared to controls and their mRNA expression and protein synthesis of *ferritin2* were persistently inhibited. These findings demonstrated that the *ferritin2* dsRNA daily ingestion not only has the effect on the long-term inhibition of mRNA expression and protein synthesis of *ferritin2* but also does not damage bees. This kind of long-term inhibition can be used for behavioral studies.

## Supporting information

S1 Raw images (PDF)

S1 Data (XLSX)
