# Supplementary material for: Long-term inhibition of ferritin2 synthesis in trophocytes and oenocytes by ferritin2 double-stranded RNA ingestion to investigate the mechanisms of magnetoreception in honey bees (Apis mellifera)
Source: PLoS One. 2021 Aug 19;16(8):e0256341. doi: 10.1371/journal.pone.0256341 (PMC8376008; doi:10.1371/journal.pone.0256341)
Supplement: S1 Raw images — (PDF) [file pone.0256341.s001.pdf]

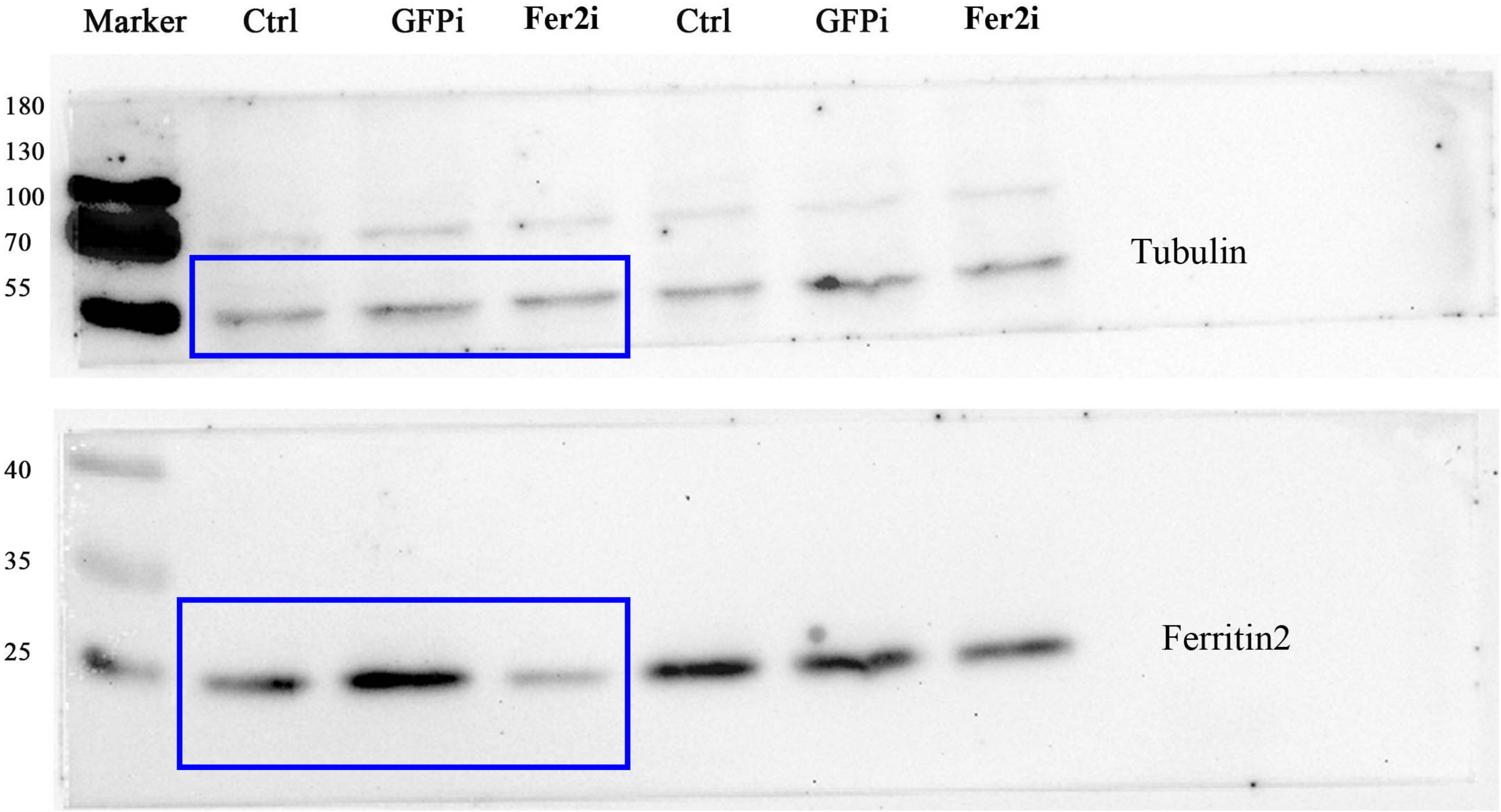

Marker
Ctrl
GFPi
Fer2i
Ctrl
GFPi
Fer2i
180
130
100
70
55
Tubulin
40
35
25
Ferritin2
